# The pathogenic exon 1 HTT protein is produced by incomplete splicing in Huntington’s disease patients

**DOI:** 10.1038/s41598-017-01510-z

**Published:** 2017-05-02

**Authors:** Andreas Neueder, Christian Landles, Rhia Ghosh, David Howland, Richard H. Myers, Richard L. M. Faull, Sarah J. Tabrizi, Gillian P. Bates

**Affiliations:** 1 UCL Huntington’s Disease Centre, Sobell Department of Motor Neuroscience, UCL Institute of Neurology, University College London, London, United Kingdom; 2 UCL Huntington’s Disease Centre, Department of Neurodegenerative Disease, Institute of Neurology, University College London, London, United Kingdom; 3 CHDI Management Inc./CHDI Foundation Inc., Los Angeles, California United States of America; 4 Department of Neurology, Boston University School of Medicine, Boston, United States of America; 5 Department of Anatomy with Radiology and Center for Brain Research, Faculty of Medicine and Health Sciences, University of Auckland, Auckland, New Zealand

## Abstract

We have previously shown that exon 1 of the huntingtin gene does not always splice to exon 2 resulting in the production of a small polyadenylated mRNA (*HTTexon1*) that encodes the highly pathogenic exon 1 HTT protein. The level of this read-through product is proportional to CAG repeat length and is present in all knock-in mouse models of Huntington’s disease (HD) with CAG lengths of 50 and above and in the YAC128 and BACHD mouse models, both of which express a copy of the human *HTT* gene. We have now developed specific protocols for the quantitative analysis of the transcript levels of *HTTexon1* in human tissue and applied these to a series of fibroblast lines and *post-mortem* brain samples from individuals with either adult-onset or juvenile-onset HD. We found that the *HTTexon1* mRNA is present in fibroblasts from juvenile HD patients and can also be readily detected in the sensory motor cortex, hippocampus and cerebellum of *post-mortem* brains from HD individuals, particularly in those with early onset disease. This finding will have important implications for strategies to lower mutant *HTT* levels in patients and the design of future therapeutics.

## Introduction

Huntington’s disease (HD) is a devastating neurodegenerative disease caused by a CAG repeat expansion in the huntingtin gene (*HTT*)^1^. Repeat expansions of 40 and above cause adult onset of the disease, with a mean age of onset of 40 years. Repeat expansions above 60–70 CAGs lead to a juvenile, much more aggressively progressive form of the disease^2^. HD presents with motor, cognitive and psychiatric symptoms, the underlying molecular basis of which is incompletely understood^2^. Model systems of HD, in particular mouse models, have been of invaluable use for researchers to study pathogenic mechanisms^3^. We recently found that in all HD knock-in mouse models, the messenger RNA of *Htt* is incompletely spliced^4^, generating a short *HTTexon1* mRNA comprised of *Htt* exon 1 and the 5′ part of intron 1, leading to the production of the exon 1 HTT protein. The R6/2 mouse line^5^, expressing precisely this fragment, is the fastest progressing HD mouse model. Furthermore, a recent study comparing the toxicity of various HTT fragments^6^ concluded that the exon 1 HTT protein is the most pathogenic HTT fragment. Interestingly, aggregates in human *post-mortem* tissue are predominantly stained with antibodies against small N-terminal fragments of HTT^7–9^. Inhibiting the production of exon 1 HTT is therefore of clinical interest and might pose a very promising strategy in treating HD.

We also found that SRSF6, a general splicing factor, tightly binds to the CAG repeat expansion^4^. This might lead to dysregulation of general splicing through local sequestration and hence depletion of this factor. Similar mechanisms lead to the molecular phenotypes of non-coding repeat expansion diseases, for example the myotonic dystrophies type 1 and 2^10, 11^. Secondary effects of dysregulation of the general splicing machinery could contribute to the extensive transcriptional dysregulation in HD patients and model systems^12–17^. Some of these, like the mis-splicing of Tau^18^, or *HTT* itself^13, 19–21^ might directly contribute to the pathogenesis of HD.

Initially, we had only limited evidence that the same *HTTexon1* mRNA was present in HD patients^4^. The extreme pathogenicity of the exon 1 HTT protein makes it a prime target for clinical intervention, and therefore, it is of uttermost importance to clarify whether this short transcript is present in patient tissue. In this study we analyzed patient derived fibroblast lines, as well as human *post-mortem* brain tissue with a wide range of CAG repeat expansions to answer this question. We developed specific protocols to quantify human *HTTexon1* transcript levels and showed that this small mRNA is indeed produced in HD patients. The levels in patient samples with juvenile onset CAG repeat ranges were highly elevated as compared to those with adult onset CAG repeat ranges and controls. We therefore conclude that the extremely pathogenic exon 1 HTT fragment is generated by incomplete splicing in a polyglutamine-length dependent manner in HD patients. This finding will have important implications for strategies to lower mutant *HTT* levels in patients.

## Results and Discussion

### *HTTexon1* mRNA is produced in patient derived fibroblast lines with large CAG repeat expansions

**Figure 1:**
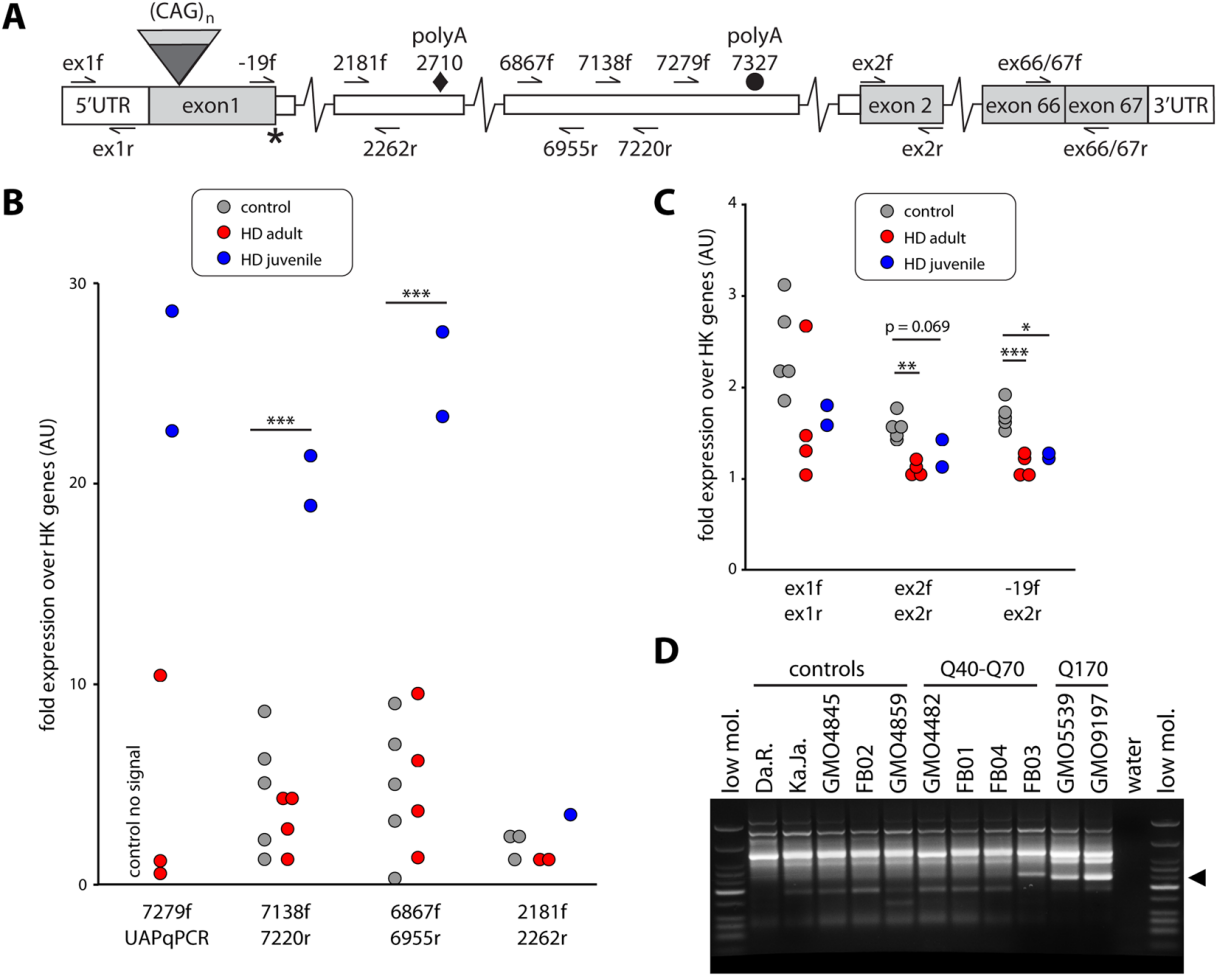
*HTTexon1* mRNA is produced in patient derived fibroblast. (**A**) Schematic representation showing the human *HTT* gene with positions and names of the qPCR assays used in this study. The cryptic polyA site at 2710 bp (◆) was only found in the YAC128 HD mouse model. The cryptic polyA site at 7327 bp (•) is the site utilized in patient samples. *The stop codon within the donor slice site. (**B** and **C**) qPCR analysis of patient derived fibroblasts. qPCR assays are detailed in Table S2. Data were grouped into control (no CAG expansion, n = 5), adult to low juvenile repeat range (HD Q40-Q70, n = 4) and large expansions (HD Q170, n = 2). For details see Table S1. Data are mean ± SEM relative to the geometrical mean of three housekeeping (HK) genes (*ATP5B*, *SDHA*, *YWHAZ*). Statistical test: ANOVA with Bonferroni post-hoc test; *p < 0.05; **p < 0.01; ***p < 0.001. (**B**) The expression level of the *HTTexon1* transcript is shown. All assays detect retention of *HTT* intron 1 sequences, for details see (**A**). UAPqPCR is homologous to the artificial sequence introduced through the reverse transcription with primer UAPdT18 (see Table S2 for primer sequences). (**C**) The expression levels of mature *HTT* mRNA transcripts are shown: exon 1 (ex1 f, ex1r), exon 2 (ex2 f, ex2r) and spliced exon 1 to exon 2 (−19f, ex2r). (**D**) A 3′RACE product (◀) was generated in fibroblast lines with large expansions of CAG repeat. low mol. = low molecular weight marker (New England Biolabs).

**Figure 2:**
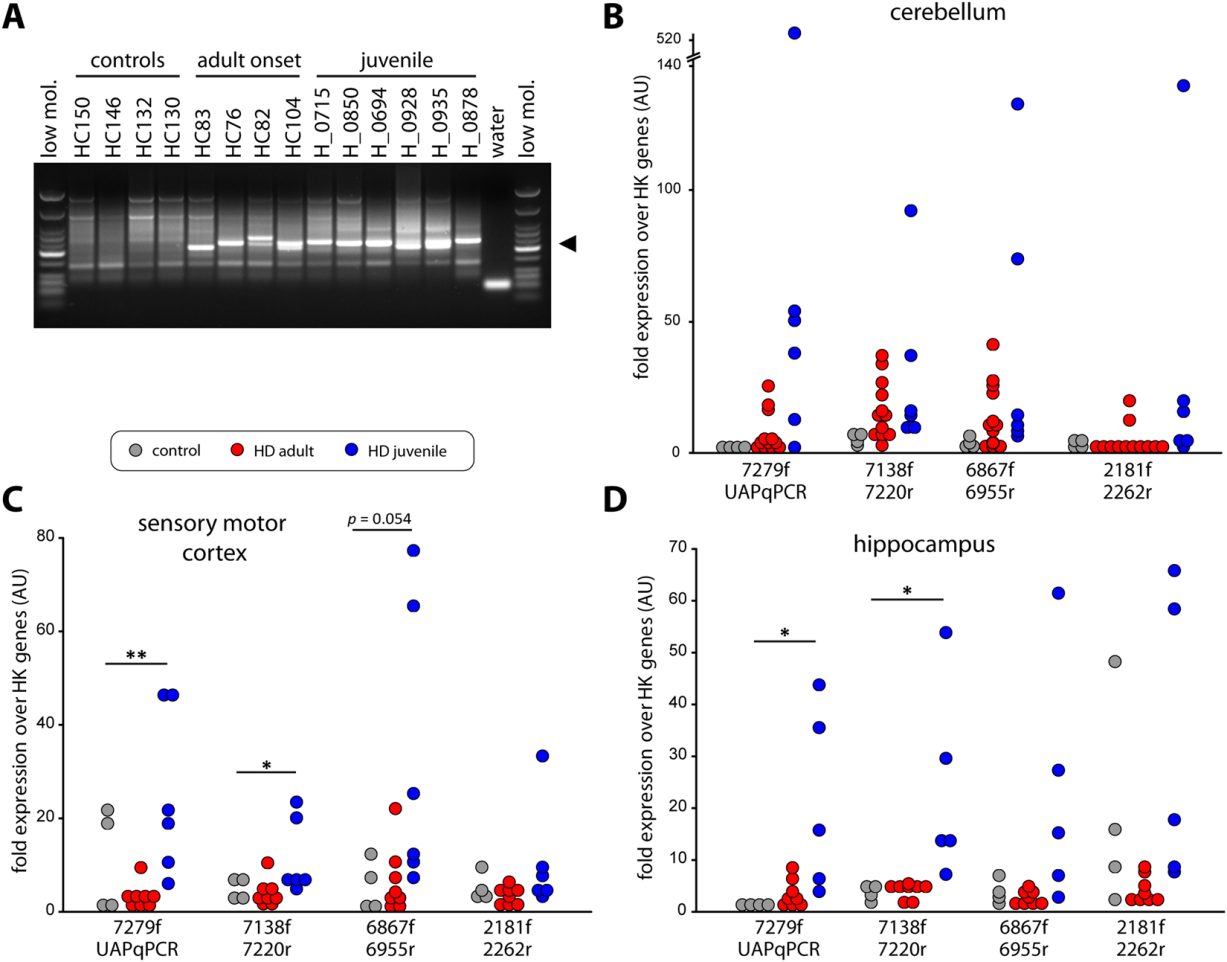
*HTTexon1* mRNA is produced in brain regions of HD patients. (**A**) 3′RACE of cerebellar extracts of *post-mortem* brain tissue from HD patients and controls. A 3′RACE product (site 7327, ◀) was generated from all samples with an expanded CAG repeat, but not from control samples. low mol. = low molecular weight marker (New England Biolabs). (**B**–**D**) qPCR analysis of cerebellum (**B**), sensory motor cortex (**C**) and hippocampus (**D**) from HD patient *post-mortem* brains. qPCR assays are detailed in Table S2. Data were grouped into control (no CAG expansion, n = 4), adult repeat range (HD adult, Q39–Q51, n = 12) and large expansions (HD juvenile, Q67-Q136, n = 6). For details see Table S1. Data are mean ± SEM relative to the geometrical mean of four housekeeping (HK) genes (*ACTB*, *ATP5B*, *SDHA*, *EIF4A2*). Statistical test: ANOVA with Bonferroni post-hoc test; *p < 0.05; **p < 0.01. qPCR assays were as described in Fig. 1.

In order to determine whether *HTTexon1* mRNA is produced in human samples, we developed a series of quantitative PCR assays (qPCR) (Fig. 1A). These were first established in a series of fibroblast lines: 5 lines without CAG expansion (control), 4 lines in the adult to low juvenile repeat range (HD Q40-Q70) and 2 lines with very large expansions (HD Q170) (Fig. 1B and C). We could readily detect the presence of the *HTTexon1* mRNA in lines with the large, juvenile onset repeat range expansions (Fig. 1B). The assays did not discriminate between the adult onset lines and controls. It is unlikely that these signals originated from heteronuclear RNA, as this would have required the oligo-dT priming to the polyA tail of the full-length *HTT* transcript and reverse transcription through the entire pre-mRNA. Alternatively, incomplete splicing may have occurred in lines from both adult onset HD and controls. These qPCR data were also reflected in the 3′RACE (rapid amplification of cDNA ends) analysis that utilized the cryptic polyA site located 7327 bp into *HTT* intron 1 (7327 site) (Fig. 1D). In line with previous results, we could not detect a 3′RACE signal for the cryptic polyA site at 2710 bp into intron 1 (2710 site), which we had found to be used exclusively in the YAC128 HD mouse model^4^. The qPCR assay, which detects *HTT* intronic sequences close to the 2710 site, located about 5 kb 5′ from the polyA tail, did not discriminate between the groups (Fig. 1B, 2181 f/2262r, see also the same assay in Fig. 2). This is consistent with the fact that the 5′ sequences of the huntingtin gene have proven to be extremely hard to analyze on numerous occasions. Next generation sequencing, as well as conventional approaches need dedicated optimization strategies to be able to detect these sequences, most probably due to the very high GC content and very stable secondary structures of the RNA^22^. In addition, the priming of the reverse transcriptase reaction from the polyA tail (UAPdT18 primer) at the 7327 site would have introduced a 3′ bias to the transcription efficiency due to the reverse transcriptases being not very processive^23^. The full-length mature mRNA was consistently expressed at a lower level in all lines carrying a CAG repeat expansion as compared to controls, independent of the length of the expansion (Fig. 1C). This phenomenon has previously been observed in HD mouse models^4^ and HD patient brains^24^. Taken together, we were able to develop quantitative assays that could detect the presence of the *HTTexon1* mRNA in fibroblast lines and furthermore, we could confirm its presence with 3′RACE analysis.

### *HTTexon1* mRNA is produced in brain tissue of HD patients

We next set out to investigate whether the *HTTexon1* transcript could be detected in *post-mortem* brains from juvenile and adult-onset HD patients and controls. To confirm the size of the large CAG repeat expansions in the juvenile onset patient samples and fibroblast lines, we performed CAG repeat sizing (Fig. S1). All samples were heterozygous for the mutated *HTT* allele. The repeat size of the non-expanded allele was in all cases between 14 and 23 CAGs as indicated by the green lines in the panels. The expanded allele for the two fibroblast lines showed maximum peak intensity at about 180 CAGs (Fig. S1, patient fibroblast lines). The expansion for the *post-mortem* brain samples varied from 67 to 136 CAGs (Fig. S1, patient brains and Table S1), confirming the juvenile onset repeat range. Interestingly, in H_0694 and H_0878, both having very large CAG repeat expansions, the peaks appeared spread out for the DNA extracted from cerebellum and sensory motor cortex (Fig. S1, H_0694 and H_0878), which could be indicative of somatic instability/expansion.

**Figure 3:**
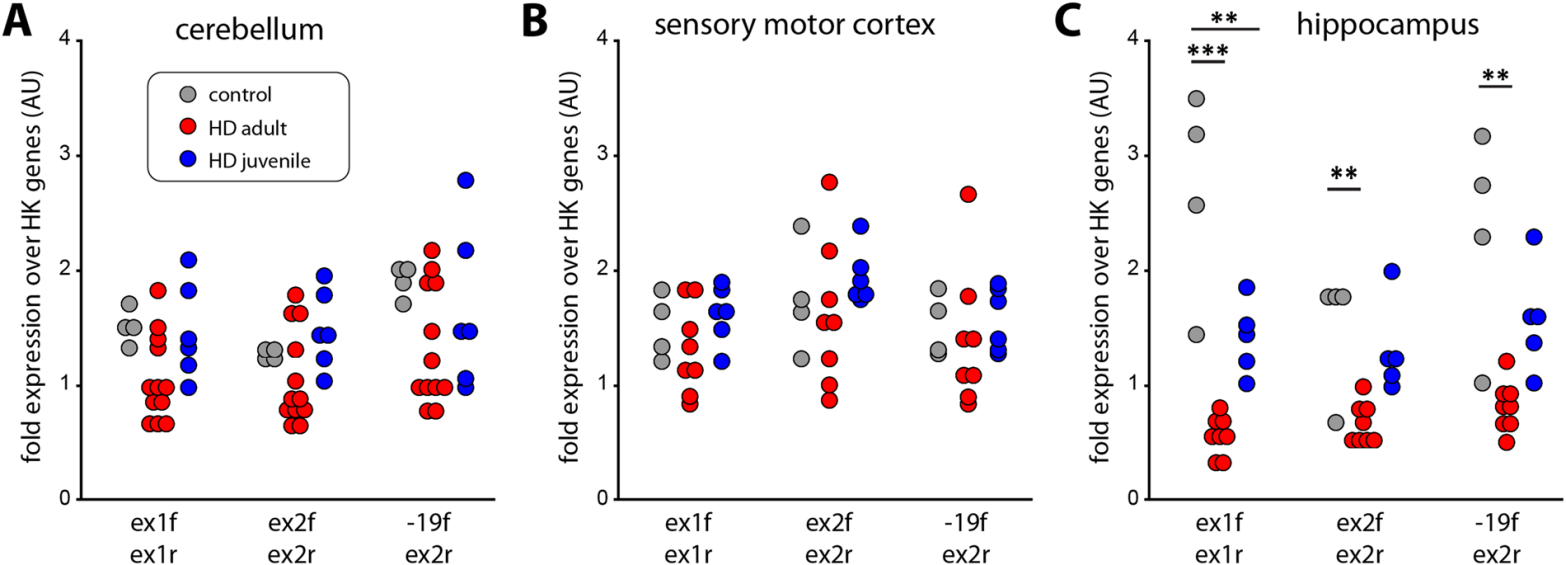
Full length *HTT* mRNA levels in brain regions of HD patients. (**A**–**C**) qPCR analysis of cerebellum (**A**), sensory motor cortex (**B**) and hippocampus (**C**) from HD patient *post-mortem* brains. qPCR assays are detailed in Table S2. Data were grouped into control (no CAG expansion, n = 4), adult repeat range (HD adult, Q39–Q51, n = 12) and large expansions (HD juvenile, Q67–Q136, n = 6). For details see Table S1. Data are mean ± SEM relative to the geometrical mean of four housekeeping (HK) genes (*ACTB*, *ATP5B*, *SDHA*, *EIF4A2*). Statistical test: ANOVA with Bonferroni post-hoc test; *p < 0.05; **p < 0.01; ***p < 0.001. qPCR assays were as described in Fig. 1.

Most brain regions from HD patients, including cerebellum, show a common transcriptional signature^15^. We used cerebellar tissue to confirm the presence of the *HTTexon1* mRNA by 3′RACE (site 7327). A product was only visible in samples with an expanded CAG repeat and not in control tissue (Fig. 2A, arrowhead). The slight difference in length of the 3′RACE products is due to the heterogeneity of oligo-dT primer binding to the polyA tail and the ambiguity of the final 3′end processing site during polyA tail synthesis^25^, combined with the very high resolution of the agarose gel. As for the fibroblast lines, we were unable to detect a 3′RACE product for site 2710. We then used our novel quantitative assays to determine the level of *HTTexon1* production in cerebellum (Fig. 2B). We could detect higher levels of *HTT* intron 1 sequences in RNA from the juvenile samples as compared to the control brains (Fig. 2B, assays 7279 f/UAPqPCR, 7138 f/7220r, 6867 f/6955r). For some samples we could detect the presence of *HTTexon1* mRNA in adult onset brain tissue, however this was more heterogeneous (Fig. 2B). To further confirm our results, we performed the same analysis on the hippocampus (Fig. 2C) and sensory motor cortex (Fig. 2D). As seen for the cerebellum, *HTTexon1* transcripts were readily detectable in the juvenile brain samples (Fig. 2C and D, HD juvenile). However, in both, hippocampus and sensory motor cortex, there was only a slightly higher signal of *HTTexon1* in the adult onset group compared to control samples (Fig. 2C and D, HD adult). Our data suggest that the *HTTexon1* transcript may be present in control as well as HD patient brains, which would be consistent with the proposed mechanism of SRSF6 binding, and lead to the production of exon 1 HTT in both cases. However, the exon 1 HTT protein with a polyglutamine tract in the normal range is not pathogenic. Analysis of full length spliced *HTT* transcripts in the cerebellum and sensory motor cortex of HD patients revealed no major differences between control and HD samples (Fig. 3A and B). In contrast, full length *HTT* mRNA was downregulated in the hippocampus (Fig. 3C), as observed in the patient derived fibroblast lines (Fig. 1C). In summary, our data clearly show the presence of the *HTTexon1* mRNA in *post-mortem* HD patient brains, largely in a CAG repeat expansion dependent manner.

### Analysis of HTT exon 1 protein levels in patient tissue and the zQ175 HD mouse model

**Figure 4:**
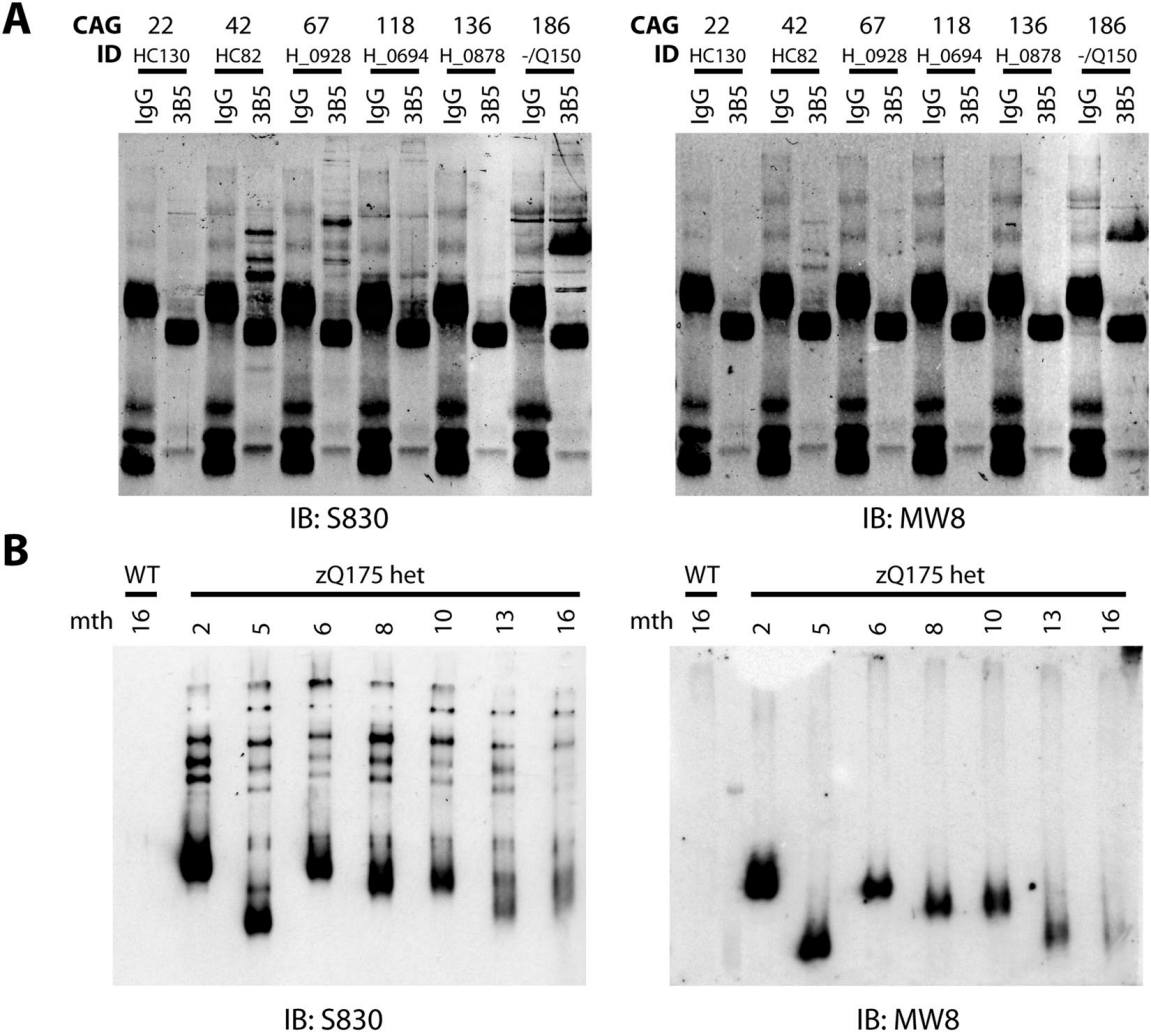
Analysis of exon 1 HTT protein in patient tissue and the zQ175 HD mouse model. (**A**) HTT proteins were immunoprecipitated with 3B5H10-dynabeads from human *post-mortem* cerebellar tissue with a wide range of CAG expansions (Q22 to Q136). Immunoprecipitation of HTT proteins from a heterozygous *Hdh*Q150 mouse brain (−/Q150) was used as a control for successful precipitation of HTT proteins. Western blots were immunoprobed with S830 (detects exon 1 HTT and larger HTT fragments) and MW8 (detects only the HTT exon 1 protein). IgG = IgG immunoprecipitation control; 3B5 = 3B5H10. (**B**) Age-dependent analysis of HTT fragments in the zQ175 mouse brains. HTT proteins from half brains of zQ175 mice at different ages (2 to 16 months (mth)) were immunoprecipitated with 3B5H10-dynabeads and western blots were immunoprobed with S830 and MW8. The observed shift in the running behavior of fragments is due to the different polyQ expansions in the individual mice. WT = wild type control with no repeat expansion.

We have previously shown that a range of N-terminal HTT fragments are present in HD patient tissue^26^. In knock-in mice, whilst the majority of these N-terminal HTT fragments are most likely generated by proteolysis, we have demonstrated that the smallest exon 1 HTT protein is the consequence of incomplete splicing. Here, we demonstrate that *HTTexon1* transcripts are also present in patient derived fibroblast lines with long CAG repeats (Fig. 1) and in the cerebellum, hippocampus and sensory motor cortex (Fig. 2) of HD patient brains. Next, we investigated whether the *HTTexon1* transcripts were translated into the exon 1 HTT protein. To this end, we immunoprecipitated HTT proteins from cerebellar extracts (Fig. 4A) and detected the precipitated proteins with S830 (detects exon 1 HTT, full length HTT, as well as other HTT fragments) and MW8 (detects only exon 1 HTT) antibodies. Cerebellar extracts with CAG repeats in the normal range were used as negative controls (Fig. 4A, HC130) and an *Hdh*Q150 heterozygous brain as a positive control, which gave a good signal for exon 1 HTT, as detected by MW8 (Fig. 4A, −/Q150). In cerebellar extracts from HD patients, we could detect HTT fragments whose intensity decreased with the increase in CAG repeat length (Fig. 4A, HC82 to H_0878, S830). However, we could not detect a clear signal for exon 1 HTT by MW8 immunoprobing (Fig. 4A, HC82 to H_0878, MW8). Given that the exon 1 HTT protein aggregates rapidly^27, 28^, we wondered whether the lack of a monomeric exon 1 HTT signal in the *post-mortem* brains was due to the fact that the exon 1 HTT protein had aggregated. Therefore, we immunoprecipitated HTT and HTT fragments at different stages of disease from the brains of zQ175 knock-in HD mice (Fig. 4B). Immunoblotting with S830 clearly showed soluble HTT fragments in the heterozygous animals but not in control brains (Fig. 4B, S830). Interestingly, the signal intensity of the overall HTT fragments decreased quite dramatically over the course of disease progression (Fig. 4A). This decrease was more pronounced for smaller fragments, with some of the larger ones still being visible at approximately the same intensity at earlier and later stages. The most dramatic decrease in signal intensity occurred for exon 1 HTT, as visualized by immunoprobing with MW8 (Fig. 4B, MW8). In summary, we found that soluble levels of exon 1 HTT diminish to a great extent during disease progression, which is consistent with the lack of signal in the human patient brains, if the exon 1 HTT protein had been recruited into insoluble aggregates.

### Implications of *HTTexon1* production in HD patients

**Figure 5:**
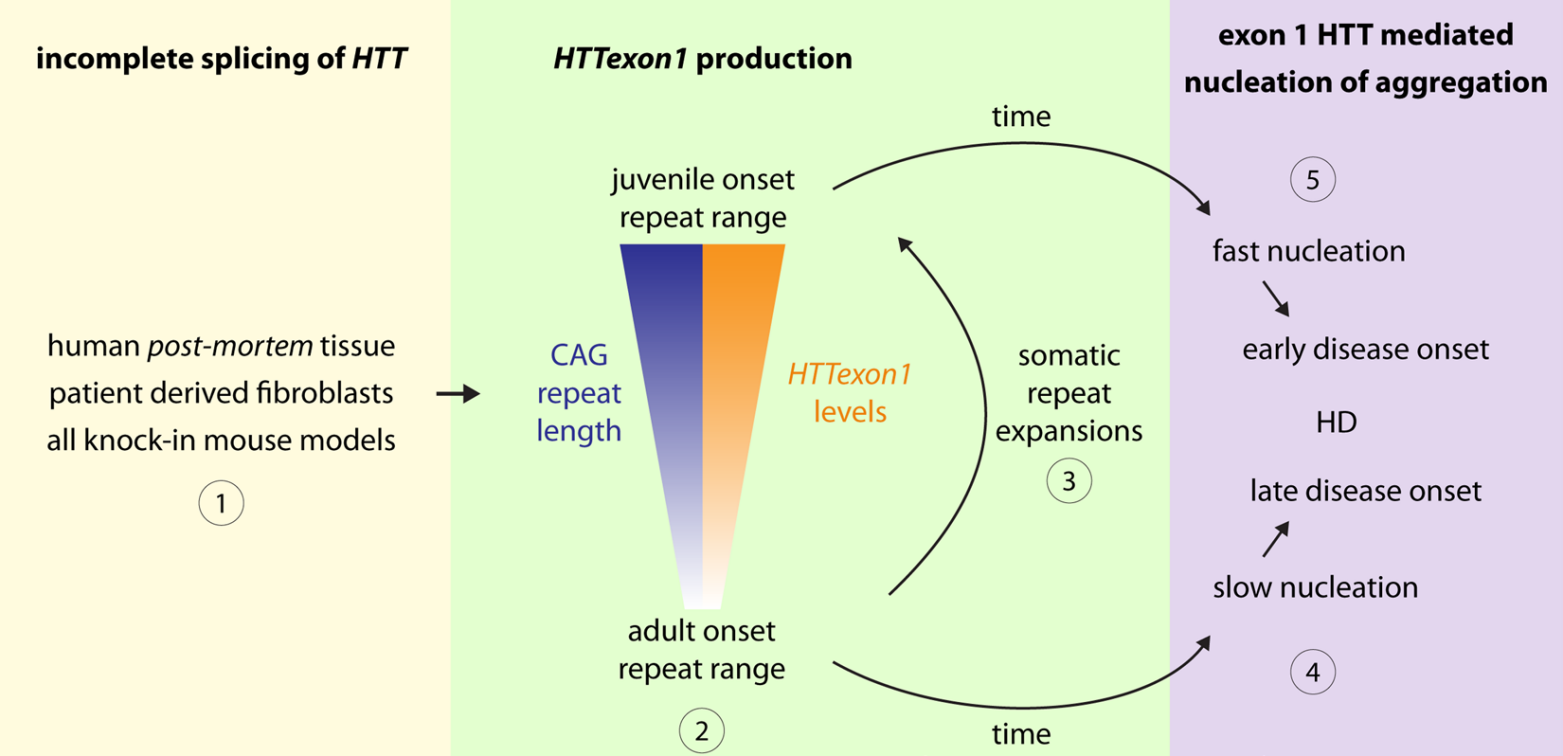
Schematic depicting incomplete *HTT* splicing and its implications for disease onset and progression. The production of the incompletely spliced *HTTexon1* message (①) is CAG repeat length dependent with longer CAG repeats resulting in higher levels of the transcript (②). The CAG repeat is unstable *in vivo* resulting in an increase in CAG repeat length (③)^31^. Exon 1 HTT levels will eventually reach the concentration threshold for nucleation of aggregation over time (④). In tissues/cell types with larger CAG expansions, which lead to higher levels of exon 1 HTT, the concentration threshold will be reached more quickly and the disease will manifest earlier (⑤).

Our finding that exon 1 HTT is produced via incomplete splicing of the *HTT* message in both HD patient tissue, as well as mouse models of HD, has several important implications for our understanding of disease pathogenesis and the design of clinical interventions (Fig. 5). The production of the *HTTexon1* message is clearly CAG repeat length dependent with longer CAG repeats resulting in higher levels of this small transcript. The *HTTexon1* mRNA is readily detectable in fibroblast cell lines (Fig. 1) and *post mortem* brain samples (Fig. 2) from patients with juvenile CAG repeat lengths. In samples with repeat lengths in the adult onset range, the levels of the *HTTexon1* mRNA were lower than in the juvenile range, and in some cases comparable to levels in control brains. The CAG repeat is unstable *in vivo* ^29, 30^ and can dramatically increase in length through somatic repeat expansion^31^. Certain tissues or cell types are more prone to somatic expansion than others and thus might have a higher disease burden^31–34^. Intriguingly, somatic repeat instability is also a good predictor of age of disease onset^35^. Although the level of *HTTexon1* transcript in tissue from adult onset brains was comparatively modest in these bulk tissue assays, this may mask higher levels in neurons in which CAG repeat expansion has occurred. We know from mouse models, that the exon 1 HTT protein is highly pathogenic and very aggregation prone. Therefore, it may only be present at relatively low levels in the tissues from HD patients with adult onset disease. It is possible that somatic expansion is required to initiate this process (Fig. 5).

Lowering the levels of *HTT* is a rational therapeutic strategy for HD and is being pursued though a variety of approaches^36, 37^. Lowering *HTT* levels through the administration of antisense oligonucleotides was well tolerated for at least 6 months in non-human primate^38^ and lowering *HTT* levels has improved many phenotypical symptoms in preclinical trials in various HD models^39–42^. Consequently, the first clinical trial to lower full length *HTT* levels in HD patients using antisense oligonucleotides was initiated in 2015 (Ionis Pharmaceuticals in collaboration with Roche; https://clinicaltrials.gov/ct2/show/NCT02519036). However, strategies that lower the levels of *HTTexon1*, as well as the full length *HTT* transcript, and thereby target the probable source of aggregate nucleation, might be expected to have an even greater therapeutic value.

## Material and Methods

### Cell line maintenance and RNA extraction

Patient derived fibroblasts were obtained from the Coriell Institute for Medical Research (lines labeled with GMO), were established in the laboratory of Prof. Sarah Tabrizi (UCL, UK) (lines labeled with FB), or were as previously described (lines Da.R. and Ka.Ja.)^43^. Lines were maintained in DMEM (high glucose, Thermo Fisher, 11960) supplemented with 20 mM L-glutamine (Thermo Fisher, 25030), 10% fetal bovine serum (Thermo Fisher, 16000) and 100 U/ml penicillin/streptomycin (Thermo Fisher, 15140). Cells were grown to about 80% confluency, detached with trypsin treatment (Thermo Fisher, 15140) for 5 min at 37 °C, pelleted for 5 min at 100 g and the pellets snap frozen in dry ice and stored at −80 °C. Cell pellets were resuspended in 100 µl DPBS (Thermo Fisher, 14190), 700 µl of QIAzol (QIAGEN, 79306) was added and mixed for at least 30 seconds until a homogenous mixture was produced. 300 µl of chloroform (VWR, 22711.244) was added, vortexed for 30 seconds and phases were separated by centrifugation at 15000 g at 4 °C. An equal volume of 70% ethanol (v/v) was added to the aqueous phase and purified with the RNeasy Mini Kit (QIAGEN, 74106). A 30 minutes genomic DNA digestion step (DNAse I, QIAGEN, 79254) was performed between the RW1 buffer washes. RNA was eluted with water and concentration was measured on a nanodrop 1000 (NanoDrop).

### Human tissue collection and RNA extraction

The use of post mortem brain samples from the New Zealand Brain Bank was approved by the St. Thomas’ Hospital Research Ethics Committee (EC03/103). The Boston University School of Medicine Institutional Review Board designated this study exempt (Protocol # H-28974), as no human subjects were studied and all data are derived from post-mortem human brain specimens. All skin biopsies were performed in accordance with the Declaration of Helsinki and approved by the NRES London – Queen Square Research Ethics Committee (LREC 03/N008, amendment 16). The subjects were recruited through the Huntington’s disease clinic at the National Hospital for Neurology and Neurosurgery, London. All subjects provided fully informed written consent. The CAG repeat sizes, postmortem delay and Vonsattel grade of the brains used in this study are summarized in Table S1.

Approximately 150 to 200 mg of human tissue was lysed in 750 µl of QIAzol (QIAGEN, 79306) in FastPrep-24 Lysis Matrix D tubes (MP BIOMEDICALS, 116913500) with a MP FastPrep-24 5 G sample preparation system (MP BIOMEDICALS) at 4 °C. 3 cycles of the following program were used with 30 seconds break in between: 7.5 m/sec speed, quickprep adapter, 60 sec, lysing matrix D, 150 mg quantity. After lysis, tubes were briefly centrifuged at 4 °C and the supernatant was transferred to a new 1.5 ml reaction tube. 300 µl of chloroform was added and the subsequent purification was performed as described above for fibroblasts.

### Reverse transcription and 3′RACE

4 µg total RNA was reverse transcribed (MMLV, Invitrogen) using the UAPdt18 primer as follows. In at total volume of 9 µl, 4 µg of RNA were mixed with 2 µl of 0.1 M dithiothreitol and 100 ng of UAPdt18 primer. The mix was heated to 95 °C for 5 min and rapidly cooled to 4 °C. 8 µl of the following mix was added: 2 µl of 10 mM dNTPs, 4 µl of 5 × 1st Strand buffer (Invitrogen, 28025-021), 0.25 µl of RNasin Plus (Promega, N261B) and 200 U of M-MLV (Invitrogen, 28025-021). The reaction was incubated for 10 min at 23 °C, 37 °C for 40 min, 94 °C for 5 min and cooled down to 15 °C. After the RT reaction, the mix was digested with 1 U of RNase H (Invitrogen) for 1 h at 37 °C. The cDNA was subsequently diluted 1:10 in water and 2 µl were used as template for the RACE PCRs. All PCRs were carried out using the Promega GoTaq system. Each PCR contained 5 µl of 5 × Green Flexi Buffer, 2 µl 25 mM MgCl2, 0.5 µl 10 mM dNTPs, each 0.5 µl of 10 mM primers, 2 µl template (cDNA or previous RACE PCR), 0.125 µl GoTaq polymerase and water to 25 µl. PCR protocols for the 3′RACE were as follows: 1^st^ and 2^nd^ 3′RACE PCR: 1 cycle 94 °C for 2 min. 30 cycles 94 °C for 20 sec, 62 °C for 20 sec, 72 °C for 1 min. 1 cycle 72 °C for 3 min followed by cooling to 15 °C. Primers were 6867 f and UAPqPCR for the 1^st^ PCR and 6987 f and UAPqPCR for the 2^nd^ PCR. 3^rd^ 3′RACE PCR: 1 cycle 94 °C for 2 min. 33 cycles 94 °C for 20 sec, 62 °C for 20 sec, 72 °C for 20 sec. 1 cycle 72 °C for 2 min followed by cooling to 15 °C. Primers were 7138 f and UAPqPCR. Primer sequences are detailed in Table S2. 3′RACE products were confirmed by sequencing to be the same as those previously described^4^.

### Human quantitative RT-PCR

3 µl of the same diluted cDNA as detailed above was used for quantitative RT-PCR (qPCR) in a 15 µl reaction mix. qPCR was carried out using the SsoAdvanced™ Universal Probes Supermix (Biorad, 1725284) and probes for references genes (PrimerDesign, HK-DD-hu-900) or custom made primer/probe sets (eurofins Genomics) as detailed in Table S2. qPCR program was as follows: 1 cycle 95 °C for 40 sec. 50 cycles 95 °C for 7 sec, 60 °C for 20 sec. Crossing thresholds (Ct) were in the range of 18 to 36 cycles. qPCR assays were run as follows: not multiplexed: *EIF4A2* (FAM), *ATP5B* (FAM), 7279f/UAPqPCR (TexasRed), −19f/ex2r (FAM), ex2f/ex2r (FAM); multiplexed: *ACTB* (FAM) & 7138f/7220r (TexasRed), *SDHA* (FAM) & 6867 f/6955r (Cy5.5), ex1f/ex1r (FAM) & 2181f/2262r (TexasRed). Evaluation was carried out with Microsoft Excel, using the 2^−ΔΔCt^ method^44^. The qPCR reaction was routinely performed on RNA (that had not been reverse transcribed) to determine the level of any signal that could have originated from genomic DNA contamination.

### Repeat sizing

A small amount (less than 10 mg) of human tissues was lyzed overnight in 300 µl of 10 mM Tris-Cl pH 8.0, 5 mM EDTA pH 8.0, 0.2% SDS, 200 mM NaCl and 0.1 mg/ml (w/v) trypsin at 50 °C. 500 µl of a isopropanol was added, briefly vortexed and incubated at room temperature for 30 min. DNA was precipitated by centrifugation at 15000 g for 15 min. The pellet was washed twice with 700 µl of 70% ethanol (v/v). After removal of residual ethanol, the pellet was resuspended in 10 mM Tris-Cl pH 8.0. Primers used were CAG1-FAM 5′-ATGAAGGCCTTCGAGTCCCTCAAGTCCTTC (5′ end is 6-carboxyfluorescein labeled) and HU3rev 5′-GGCGGCTGAGGAAGCTGAGGA. Repeat sizing PCR mix was as follows: 60 ng DNA, 1 µl of 2 mM dNTP mix, 7.2 µl of 5.5 M betaine, 2 µl of PCR buffer (300 mM Tris-Cl pH 8.9, 160 mM ammonium sulfate, 25 mM magnesium chloride, 1.5 mg/ml (w/v) bovine serum albumin, 1 mM β-mercaptoethanol), 2 µl of each 10 µM primer, 0.2 µl Herculase (Agilent Technologies 600264). PCR program was as follows: 1 cycle 95 °C for 5 min. 32 cylces of 94 °C for 30 sec, 60 °C for 30 sec and 72 °C for 3 min. 1 cycle of 72 °C for 5 min followed by cooling down to 15 °C. 1 µl of the PCR product was denatured at 95 °C for 5 min followed by rapid cooling to 4 °C in 9 µl HiDi formamide (Applied Biosystems, 4311320) supplemented with MapMarker 1000 Rox 1000 size standard (BioVentures, MW-0195-80ROX). The repeat sizes were run on a 3730xl DNA Analyzer (Applied Biosystems) and analyzed with the GeneMarker software (SoftGenetics).

### Mouse maintenance, breeding and genotyping

*Hdh* ^Q150/Q150^ homozygous, *Hdh* ^+/Q150^ heterozygous mice and wild type littermates on a (CBA × C57BL/6) F1 background were obtained by intercrossing *Hdh* ^+/Q150^ heterozygous CBA/Ca and C57BL/6 J congenic lines as described previously^45^. The zQ175^46^ knock-in mice were supplied from CHDI colonies maintained at The Jackson Laboratory (Bar Harbor, ME, USA). The zQ175 lines were maintained by backcrossing to C57BL/6J (Charles River) and homozygotes, heterozygotes and wild type littermates were generated by intercrossing as required. All experimental procedures performed on mice were approved by the University College London Ethical Review Process Committee and carried out under a Home Office License. All animals had unlimited access to food and water, were subject to a 12-h light/dark cycle and housing conditions and environmental enrichment were as previously described^47^. Genomic DNA was isolated from an ear-punch. *Hdh*Q150 mice were genotyped by PCR and the CAG repeat length was measured as previously described^48^. The genotyping primers for zQ175 were as in ref. 46 using the R6/2 genotyping protocol^48^. Dissected tissues were snap frozen in liquid nitrogen and stored at −80 °C until further analysis.

### Mouse quantitative RT-PCR

RNA extraction, reverse transcription and quantitative RT-PCR (qPCR) for mouse samples were performed as previously described^4^. RNA was reverse transcribed from an oligo-dT primer (UAPdT18, Table S2) and qPCR was performed in the same way as for the human tissue samples as detailed above. Primers and probe sets are described in detail in ref. 4.

### Antibodies, immunoprecipitation and western blotting

3B5H10 is a monoclonal antibody that was raised against an N-terminal 171 amino acid fragment of HTT with 65Q and detects a polyQ tract^49^ (Sigma), S830 is a sheep polyclonal antibody raised against exon 1 HTT with 53Q^50^ and MW8 is a monoclonal raised against the peptide AEEPLHRP at the C-terminus of exon 1 HTT^51^. 3B5H10 coupling to magnetic beads (Dynabeads M-270 Epoxy; Invitrogen) was performed as described in ref. 4. The same 3B5H10-dynabeads conjugate was used to immunoprecipitate HTT from human tissue. 200 mg of human tissue was lyzed in 800 µl of 50 mM HEPES pH 7.6, 160 mM NaCl, 10 mM EDTA pH 8.0, 1% (w/v) Triton X-100, 0.1% (w/v) SDS, 0.2% (w/v) sodium deoxycholate, 2 mM dithiothreitol, 0.1% (v/v) PMSF, protease inhibitors (Complete Mini, Roche, 11836170001) using the same lysis procedure as described above for RNA extraction of human tissue. Lysates were cleared by 2 consecutive centrifugation steps at 15000 g at 4 °C for 15 min each. 15 µl of pre-washed 3B5H10-dynabeads were added to 3 mg (BCA assay, Thermo Scientific, 23225) of cleared lysate and topped up to a final volume of 1 ml with lysis buffer. The immunoprecipitation (IP) was incubated overnight at 4 °C with constant motion on a rotating wheel. The IP was washed four times with 0.5 ml of lysis buffer at room temperature. Captured proteins were eluted in 20 µl of 200 mM Tris-Cl pH 6.8, 1 mM EDTA pH 8.0, 5% (w/v) SDS, 215 mM β-mercaptoethanol, 8 M urea and incubated for 15 min at 65 °C. Western blotting and immunoprobing were performed as previously described^52^. Signals were visualized on an Odyssey Sa Imaging System (LI-COR Biosciences). Immunoprecipitation, western blotting and immunoprobing for mouse tissue were performed as previously described^52^.

### Statistics

Statistical significance was calculated by one-way or two-way ANOVA with the Bonferroni post-hoc test.

## Electronic supplementary material

Supplementary info
