# Supplementary material for: The pathogenic exon 1 HTT protein is produced by incomplete splicing in Huntington’s disease patients
Source: Sci Rep. 2017 May 2;7:1307. doi: 10.1038/s41598-017-01510-z (PMC5431000; doi:10.1038/s41598-017-01510-z)
Supplement: Supplementary file 1 — Supplementary info [file 41598_2017_1510_MOESM1_ESM.pdf]

The pathogenic exon 1 HTT protein is produced by incomplete splicing in Huntington's disease patients

Andreas Neueder[1], Christian Landles[1], Rhia Ghosh[2], David Howland[3], Richard H. Myers[4], Richard L. M. Faull[5], Sarah J. Tabrizi[2] and Gillian P. Bates[1]*

[1]UCL Huntington's Disease Centre, Sobell Department of Motor Neuroscience, UCL Institute of Neurology, University College London, London, United Kingdom

[2]UCL Huntington's Disease Centre, Department of Neurodegenerative Disease, Institute of Neurology, University College London, London, United Kingdom

[3]CHDI Management Inc./CHDI Foundation Inc., Los Angeles, California, United States of America

[4]Department of Neurology, Boston University School of Medicine, Boston, United States of America

[5]Department of Anatomy with Radiology and Center for Brain Research, Faculty of Medicine and Health Sciences, University of Auckland, Auckland, New Zealand

*To whom correspondence may be addressed. E-mail: gillian.bates@ucl.ac.uk. Phone: +44 (0)20 3448 4522.

SUPPLEMENTARY ITEMS

Supplementary Figures

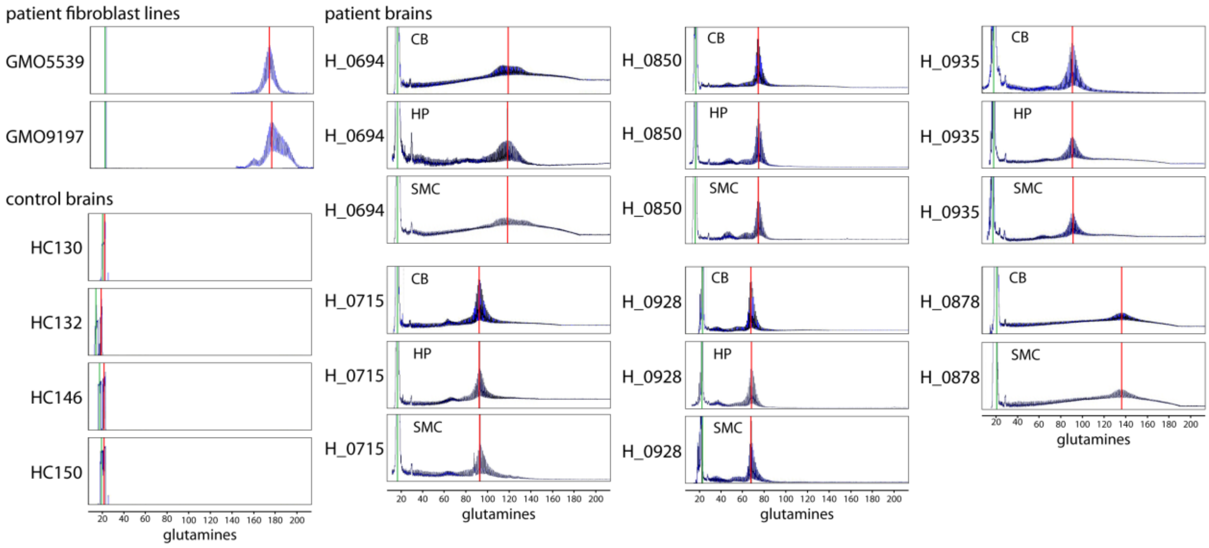

Figure S1. CAG repeat sizes of samples in the juvenile onset repeat range. Samples were repeat sized as detailed in the Material and Methods section. Patient derived fibroblast lines and brain tissue used in previous studies are described in Table S1. The red vertical line highlights the maximum intensity of the larger allele, the green line of the allele without repeat expansion. CB = cerebellum; HP = hippocampus; SMC = sensory motor cortex.

Supplementary Tables

Table S1. Details of human fibroblast lines and human *post-mortem* brain tissue.

Tissue	Identification	Q-length	*Post-mortem* delay [h]	Vonsattel Grade
Fibroblasts	Da.R.	16/16		
	Ka.Ja.	16/16		
	GMO4845	16/20		
	GMO4859	16/20		
	GMO4482	20/45		
	GMO5539	21/177		
	GMO9197	21/180		
	FB01	22/55		
	FB02	21/21		
	FB03	22/71		
	FB04	22/65		
Brain	HC130	20/22	13	
	HC132	14/19	12	
	HC146	17/21	15	
	HC150	19/21	12	
	HC103	19/39	11	1
	HC83	20/40	9	1
	HC74	17/42	11	1
	HC105	15/42	9	1
	HC72	17/42	24	2
	HC76	19/42	16	2
	HC82	15/42	16	2
	HC102	17/42	10	3
	HC58	18/44	19	3
	HC85	24/44	19	3
	HC73	19/49	4	2
	HC104	18/51	15	3
	H_0928	23/67		4
	H_0850	16/74		4
	H_0935	18/90		3
	H_0715	16/92		3
	H_0694	16/118		4
	H_0878	20/136		3

Table S2. Details of primers and probes used in this study.

Name	Internal name	Sequence 5'→ 3'	Probe dye/quencher	Position from start of HTT intron 1; start/end in bp
ex1f	hex1for	GGGTCCAAGATGGACGGC		5' UTR
ex1r	hex1rev	AGCACCGGGGCAATGAATG		5' UTR
ex1p	hex1p	CAGGTTCTGCTTTTACCTGCGGCC	FAM/TAMRA	5' UTR
ex2f	hex2for	AAAGAAAGAACTTTCAGCTACCAAGAA		exon 2
ex2r	hex2rev	CTGACAGACTGTGCCACTATGTTT		exon 2
ex2p	hex2p	ACCGTGTGAATCATTGTCTGACAATATG	FAM/TAMRA	exon 2
-19f	mHtt--19f	AGGAACCGCTGCACCGA		-19/-3
2181f	3124	ATCTTCCTTCCACCCCTTTCC		2181/2201
2262r	2676r	GGCAGGATACATGAGGAACTG		2262/2242
2239p	2622p	ATAGTGCTTGTTCTTGGGGACAAAGTTAGGTA	TexasRed/BHQ2	2239/2208
6867f	7281	AGCACAGATGAAAAACAAAGCCCT		6867/6890
6955r	7369r	GGGAAGAAGACACAAGAAACACTT		6955/6932
6893p	7307p	TTGCAAGTCTGTCATCTTTGTCTAACTTCCTA	Cy5.5/BHQ3	6893/6924
7138f	7552	GGAGATGTAAAGGCAAAGGTCTC		7138/7160
7220r	7664r	TGGGGACTTCTCAAGGTAGAGA		7220/7199
7163p	7577p	ATTTTTGTGGCTGTAGAATGTGCTGGTGAC	TexasRed/BHQ2	7163/7192
7279f	7693	GGATCCACACTCAAAACATTTA		7279/7300
7302p	7716p	GTCTTATTCAGACAACAAGGAGGAAAAATAAAATACC	TexasRed/BHQ2	7302/7337
6987f	7401	TGTGTCATTTTTTGTTTGCTTGACTGA		6987/7013
UAPqPCR	UAPqPCR	CACGCGTCGACTAGTAC		
UAPdT18	UAPdT18	GGCCACGCGTCGACTAGTACTTTTTTTTTTTTTTTTTT		
